# Text Message (SMS) Microlearning for Tobacco Use Disorder: Pre-Post Pilot Study of Clinician Confidence

**DOI:** 10.2196/73821

**Published:** 2025-12-09

**Authors:** Zehra Dhanani, Veena Dronamraju, Jamie Garfield

**Affiliations:** 1 Thoracic Medicine and Surgery, Temple University Hospital, 3401 N Broad St, Philadelphia, PA, 19140, United States, 1 800-836-7536; 2 Department of Lung and Respiratory Care, Mount Auburn Hospital, Cambridge, MA, United States; 3 Thoracic Medicine and Surgery, Lewis Katz School of Medicine, Temple University, Philadelphia, PA, United States

**Keywords:** tobacco use disorder, clinical education, text messaging, confidence, implementation, feasibility

## Abstract

**Background:**

Clinicians are central to treating tobacco use disorder, yet practical training is inconsistent, and confidence varies. Brief, text message–based microlearning may offer a low-burden way to strengthen foundational competencies in busy clinical settings.

**Objective:**

This paper aims to evaluate whether a short SMS microlearning series improves clinicians’ self-reported confidence in managing tobacco use disorder.

**Methods:**

We conducted a single-arm, pre-post educational pilot at an academic medical center. A brief formative survey (13 items; 106 respondents) identified local knowledge gaps and informed message topics and sequencing. The 13-day series delivered 1 concise message per day with key teaching points and links to curated resources. The prespecified primary outcome was self-reported confidence in managing tobacco use disorder (1‐100 scale) measured immediately before and after the series. Of the 34 clinicians who signed up, 22 completed the baseline questionnaire and enrolled (attendings: n=4, 18%; trainees: n=18, 82%). Changes in confidence among participants with paired ratings were tested with a paired *t* test. Engagement with embedded links was recorded.

**Results:**

All enrolled participants completed the 13-day series; none unsubscribed. Postintervention confidence ratings were provided by 18 participants. Mean confidence increased from 60 (SD 16) at baseline to 85 (SD 10) after the series (*t*_17_=−10.71; *P*<.001). Embedded links were opened in 67% (178/266) of messages. Free-text feedback was predominantly positive and emphasized the convenience, clarity, and point-of-care usefulness of brief messages.

**Conclusions:**

A brief SMS microlearning series was associated with a substantial improvement in clinicians’ confidence to manage tobacco use disorder, with high completion and evidence of engagement. This low-cost, scalable approach appears practical for busy clinicians. Findings should be interpreted cautiously given the single-arm design, self-selection, and reliance on self-reported confidence rather than objective knowledge or clinical outcomes. Future studies should include a validated knowledge assessment, a randomized comparison, broader sampling, and follow-up to assess durability and impact on care.

## Introduction

Tobacco use remains the leading cause of preventable mortality in the United States, accounting for nearly 20% of all deaths [1]. In addition to its negative health implications, smoking imposes significant economic burdens on the health care system [2]. Health care professionals are tasked with the critical responsibility of managing tobacco use disorder and its associated morbidities. However, data suggest that there are substantial knowledge gaps among physicians. In a United States survey of health care providers, fewer than half demonstrated high knowledge of availability of diagnostic criteria (36.8%), treatment efficacy (33.2%), counseling modalities (5.6%), and US Food and Drug Administration–approved medications (40.1%) [3]. Similar gaps are reported internationally; among primary care physicians in 3 Malaysian districts, 62.4% had poor knowledge, 58.0% had poor attitude, and practice was poor for 50.9% in the precontemplation phase and 75.7% in the contemplation phase of smoking-cessation management [4]. These deficits likely reflect uneven training exposure; evidence indicates that education in tobacco use disorder treatment is often inadequate, competency assessments are infrequent, and the topic receives minimal attention in board certification examinations [4-6].

At our institution, we conducted a brief formative survey to identify local gaps in knowledge and confidence and used these findings to shape the content and pacing of the intervention; full methods and the survey instrument are provided in the Methods and Multimedia Appendix 1.

Based on the results of survey data and identified knowledge gaps, we sought innovative and effective methods to educate practitioners on tobacco dependence treatment. Our objective was to identify learning strategies that were not time-consuming, easy to understand, readily accessible, and sustainable. Given these aims, we selected text message–based microlearning, which delivers brief, focused content that can be revisited at the point of care and reinforced over time, with demonstrated effectiveness in clinical education [7,8].

Among digital options, SMS pushes concise content without requiring app downloads or logins. It reaches busy clinicians, supports repeated exposure, and adds minimal burden, which fits the training and time constraints that healthcare providers face. We developed a brief text-based educational module to evaluate the effectiveness of this modality in teaching. Specifically, we used tobacco dependence treatment as a case example to assess whether a concise SMS series could address provider knowledge gaps and improve management confidence. Our primary hypothesis was that clinicians who completed the SMS series would report higher confidence in managing tobacco use disorder compared with baseline.

## Methods

### Study Design

We conducted a single-arm, pre-post educational pilot at an academic medical center. We designed a 13-day text message series for health care providers on tobacco dependence treatment. Concise daily messages were generated based on the knowledge gaps identified in the survey. Participation in the text-based series was completely voluntary. Surveyed participants were invited to opt in and had the option to opt out at any time. By opting in and providing their contact information, participants explicitly consented to participate in the study and receive text messages.

### Participants and Recruitment

A formative needs assessment was conducted prior to the intervention to characterize local knowledge gaps and baseline confidence. Using institutional distribution lists, we contacted 209 clinicians—including internal medicine residents, family medicine residents, internal medicine faculty, pulmonary and critical care faculty, and pulmonary or critical care fellows and advanced practice providers—to complete the survey; 106 clinicians responded. Recruitment for the SMS intervention occurred in the subsequent academic cycle and targeted a pool that was not identical to the survey cohort. Using institutional distribution lists, we reached 142 residents, 39 faculty, and 28 fellows or advanced practice providers and invited them to enroll. Opt-ins and completion numbers are reported in the Results.

### Formative Needs Assessment (Survey)

Before developing the curriculum, we administered a brief survey to characterize local knowledge gaps and baseline confidence. A total of 106 clinicians participated. The survey comprised 13 items: one 1‐100 visual analog scale for self-reported confidence in managing tobacco use disorder (1=no confidence; 100=complete confidence) and 12 knowledge items covering counseling approaches, cognitive behavioral strategies, first-line pharmacotherapy, and management in special populations (eg, pregnancy). The instrument was created for this study based on guideline topics and content domains targeted for the messages; the full survey is provided in Multimedia Appendix 1. Overall, 70% (74/106) of respondents reported feeling confident managing tobacco use disorder on the 1‐100 scale. Item-level accuracy is summarized in Table 1 (formative needs assessment informing curriculum design). Notably, only 32% (n=34) correctly identified the first-line pharmacologic treatment, and additional gaps were observed across counseling approaches, cognitive behavioral strategies, and management in pregnancy. These descriptive findings were used solely to inform topic selection and sequencing for the 13-day series and are not analyzed as study outcomes.

**Table 1.** Performance on formative survey assessing knowledge of tobacco use disorder management (n=106). Values are number and percentage of respondents who answered each item correctly.

Knowledge tested	Correctly answered, n (%)
Recommended first-line pharmacological treatment for tobacco use disorder	34 (32)
Understanding of bupropion’s contraindications	61 (58)
Nicotine patch dosing	41 (39)
Nicotine gum dosing	67 (63)
Correct technique to use nicotine gum	70 (66)
Treatment for precontemplative patients	15 (14)
Prescription of varenicline in patients with psychiatric conditions	51 (48)
Duration of varenicline treatment after quitting	61 (58)
Risk of adverse cardiovascular events with treatment in stable coronary artery disease	86 (81)
Pharmacologic interventions for smoking cessation during pregnancy	56 (53)

### Intervention

Participants who opted in received a message once every 24 hours. Each text message began with the mean performance on the survey of the particular teaching point in question, provided information or management recommendations, and concluded with live links to reference materials and additional reading for those seeking to deepen their understanding (Multimedia Appendix 2). We used a commercial SMS texting platform called SimpleTexting, a secure platform that complies with industry standards for privacy. This platform enabled the efficient dissemination of messages while protecting participants’ personal information. It also provided functionalities to monitor engagement with the provided links, receive participant responses, and track subscription status. At the start and end of the series, we assessed participants’ confidence with managing tobacco use disorder, defined as their subjective confidence on the topic, using a scale of 1 to 100. Additionally, we invited participants to provide comments and feedback on the text message series to gain qualitative insights into their experiences and perceptions. All submitted feedback was reported as received.

### Statistical Analysis

All available pre-post confidence pairs were analyzed; optional feedback was summarized descriptively and reported as received. A paired *t* test was used to compare confidence levels before and after the series, and the analysis was conducted using IBM SPSS version 25. A *P* value of less than .05 was considered statistically significant.

### Ethical Considerations

This study was approved by the Temple University Hospital Institutional Review Board (protocol 32174). Participation in both the formative survey and the SMS series was voluntary; responding to the survey and opt-in enrollment for the series constituted informed consent for receipt of educational messages and analysis of deidentified data. Messages were delivered via a commercial SMS platform (SimpleTexting); no protected health information was collected. Survey and evaluation data were deidentified and stored on secure institutional servers with aggregate reporting. Participants received no compensation. No images identifying individual participants are included.

## Results

A total of 34 individuals signed up for the series, with 22 completing the initial questionnaire on their confidence treating tobacco use disorder and subsequently enrolling in the intervention. The participants included providers at all levels of training, including attending physicians (n=4, 18%) and graduate medical education trainees (n=18, 82%) (Table 2). Before the intervention, the average confidence level in managing tobacco use disorder across all training levels was 60 (SD 16). All enrolled participants completed the series, and there were no unsubscribes. At the conclusion of the series, we received responses on confidence level from 18 participants along with comments and feedback. The average confidence level increased to 85 (SD 10). The paired *t* test revealed a statistically significant difference in confidence levels before and after the series (*t*_17_=−10.71; *P*<.001).

The reference links provided in each message were accessed 67% (178/266) of the time, indicating active engagement with the additional resources. Feedback from the participants was predominantly positive. Common themes included appreciation for the concise and informative nature of the text series and the ease of learning they provided. One participant noted, “Text-based format made it easy to learn in an efficient manner,” while another remarked, “This series absolutely increased my comfort level with treating tobacco use disorder.” Suggestions for improvement included adding visual aids (Textbox 1).

**Table 2.** Breakdown of individual participant feedback from the text-based education series.

Subject	Department	Level of training	Preintervention confidence score	Postintervention confidence score
1	IM^a^	1	50	70
2	PCCM^b^	4	50	70
3	PCCM	4	40	75
4	IM	2	30	No response
5	PCCM	5	80	100
6	IM	1	25	75
7	PCCM	5	40	80
8	PCCM	5	83	100
9	PCCM	6	60	85
10	PCCM	6	75	90
11	PCCM	4	50	85
12	IM	3	65	No response
13	IM	2	70	No response
14	IM	2	60	90
15	PCCM	6	55	90
16	PCCM	4	60	85
17	IM	3	75	No response
18	PCCM	Attending	47	70
19	PCCM	6	75	90
20	PCCM	Attending	80	90
21	PCCM	Attending	60	90
22	PCCM	Attending	75	95

a IM: Internal Medicine.

b PCCM: Pulmonary and Critical Care Medicine.

Textbox 1. **Feedback from participants and those who completed the text-based education series.**

“Text-based format made it easy to learn in an efficient manner.”

“This series absolutely increased my comfort level with treating tobacco use disorder.”

“I pinned the text chain and went back to it when I had a question.”

“The texts are awesome—very informative, short, and to the point.”

“Add visual aids next time! Otherwise, no critique.”

“Made me realize I was not using the correct dosing for nicotine! Thanks for teaching.”

“This is so innovative! Don’t stop, do another topic next!”

## Discussion

This study aimed to examine whether SMS microlearning can strengthen clinicians’ confidence in managing tobacco use disorder. Our findings indicate a meaningful increase in physician confidence after completing the intervention, and participants consistently described the messages as clear, concise, and useful at the point of care. Although these results come from a small cohort, the approach appears feasible for broader implementation and may serve as an effective educational tool; its impact on knowledge and clinical outcomes should be evaluated in larger, comparative studies.

Tobacco use remains a leading driver of preventable morbidity and mortality, and clinicians are central to diagnosis, counseling, and pharmacotherapy [9,10]. Yet tobacco treatment is underrepresented in many training programs [6,11-14], and persistent knowledge gaps have been documented across diagnostic criteria, counseling approaches, and evidence-based medications in diverse settings [3,6,13]. Tobacco treatment also receives limited emphasis on medical licensing and board examinations, which can reduce curricular time and formal assessment [5]. We observed similar gaps locally in our formative survey, which is why we selected tobacco use disorder as the initial focus for this educational pilot. Targeting a high-impact, common condition with clear guideline-based management made it possible to craft concise teaching points and to judge whether a brief intervention could realistically support everyday practice.

The realities of clinical work limit traditional didactic learning. Long shifts, competing patient care and administrative demands, and variable schedules make it difficult to attend scheduled sessions or complete lengthy modules, and even when completed, retention can fade without reinforcement [15-17]. In this context, brief touchpoints that can be completed between tasks and revisited at need are more likely to fit daily workflow. Microlearning and spaced relearning operationalize this idea by delivering small, focused units with planned repetition, promoting retrieval when it matters and supporting transfer to practice [18-20]. For clinicians managing tobacco use disorder, this approach aligns with how information is actually used at the point of care and helps convert passive exposure into durable, actionable knowledge.

SMS microlearning aligns with clinical workflow: it is low friction, works on any device, and is easy to consume between tasks. Short, focused messages support spacing and retrieval, while links allow quick escalation to peer-reviewed resources at the point of care. Evidence from health professions education suggests this format is effective and well received: multicenter and randomized studies in obstetrics and gynecology residents report improved test performance and higher learner interest with SMS curricula, along with high satisfaction and low cost [21,22]; clinicians describe SMS teaching as engaging and practice-informing [23]; and related smartphone-based microlearning projects show strong acceptability and ease of use [24]. Not all findings are uniformly positive; some trials show no long-term advantage in exam scores without integration into broader study routines [25], which underscores the need for comparative designs and objective outcomes. In our pilot, the goal was to strengthen confidence rather than to test knowledge, and our results align with the broader literature on feasibility and acceptability. These considerations motivate a careful appraisal of limitations, including the absence of objective knowledge outcomes and comparative designs.

Our study has several limitations. It was a single-arm, pre-post pilot that evaluated self-reported confidence as the prespecified primary outcome. We did not administer an objective knowledge test; the preintervention survey was a formative needs assessment to guide content rather than a validated outcome instrument. Without a comparison group, we cannot separate the effect of message content from nonspecific influences such as measurement reactivity, social desirability, or Hawthorne effects. Selection bias is likely because participation was voluntary and only a subset of enrollees provided paired ratings, which limits generalizability beyond motivated clinicians at a single academic center. The sample was small, we assessed outcomes only immediately after the series, and engagement was measured by link click-throughs, which are a proxy and do not confirm content review or practice change. Optional free-text feedback may overrepresent highly engaged participants. Results may not generalize to settings without reliable SMS access or to clinicians with different baseline training, and the study was not powered for subgroup comparisons. Future work should include a validated knowledge assessment aligned to the message set, randomization to alternative content or wait-list control, larger and more diverse samples with strategies to reach less-engaged clinicians, and follow-up to assess durability and clinical impact.

In conclusion, a brief text message–based microlearning series was associated with a substantial increase in clinicians’ confidence to manage tobacco use disorder, with strong completion and evidence of engagement. The format was practical and acceptable for busy clinicians, offering concise, point-of-care reinforcement. These results should be interpreted cautiously given the single-arm design, self-selection, and reliance on self-reported confidence rather than objective knowledge or clinical outcomes. Future studies should incorporate a validated knowledge assessment, a randomized comparison, broader sampling, and follow-up to evaluate durability and impact on patient care.

## Supplementary material

10.2196/73821 Multimedia Appendix 1 The survey used in this study that was specifically created for this research.

10.2196/73821 Multimedia Appendix 2 Text messages and references sent to the participants.
