# Supplementary material for: Text Message (SMS) Microlearning for Tobacco Use Disorder: Pre-Post Pilot Study of Clinician Confidence
Source: JMIR Med Educ. 2025 Dec 9;11:e73821. doi: 10.2196/73821 (PMC12688373; doi:10.2196/73821)
Supplement: Multimedia Appendix 1 [file mededu-v11-e73821-s001.pdf]

Smoking Cessation:

We are conducting a study to see how our providers approach smoking cessation. If you could please take a few minutes to complete this survey to the best of your ability, we would greatly appreciate it. Thank you!

Which field of medicine are you in?

- ◯ Pulmonary
- ◯ Internal Medicine
- ◯ Family Medicine

What is your current position?

- ◯ Intern
- ◯ Resident
- ◯ Fellow
- ◯ Attending
- ◯ Advanced Practice Provider

How confident do you feel treating tobacco use disorder/cessation?
(Click and drag the white circle across the bar)

Confidence

QuestionPro

How often do you counsel your patients who smoke cigarettes on smoking cessation? (Click and drag the white circle across the bar)

Frequency

How long do you spend counseling your patients on smoking cessation per visit?

- Less than 3 minutes
- Greater than 3 minutes

In addition to counseling, do you recommend any pharmacological treatments to a patient who smokes and has no contraindications to medications?

- I start with counseling alone
- Single nicotine replacement agent
- Long and short acting nicotine replacement therapy
- Varenicline
- Bupropion
- Varenicline and a nicotine patch

How likely are you to arrange a follow-up appointment specifically for smoking cessation after starting treatment?

Likelihood

Stabros is a current smoker and wants to quit. He heard about Bupropion on the radio and comes to you to learn more. He has a history of seizure disorder, depression and GERD. He was previously taking Selegiline for his depression, but stopped 6 months ago due to intolerance.

You tell Stabros that Bupropion cannot be started for him for the following reason:

- ◯ His seizure disorder
- ◯ His recent use of Selegiline (MAO inhibitor)
- ◯ All of the above

Laurie smokes 6 cigarettes a day and is interested in a nicotine replacement patch.

What dose should you prescribe?

- ◯ 7 mg
- ◯ 14 mg
- ◯ 21 mg

Naila is not ready to quit smoking cigarettes ("pre-contemplative") when you counsel her on cessation.
What should you do?

- ◯ Schedule a 1 month follow up visit to readdress the topic as Naila is not ready to quit smoking1
- ◯ Start Varenicline at this visit

Hiroshi is a long-term smoker and is interested in quitting. 3 years ago, he had suicidal ideation, however, has been working with his psychiatrist and has had a stable regimen for the last year. He does not currently have any suicidal or homicidal ideation.

What do you tell him about starting therapy?

- ◯ You recommend starting Varenicline as it has been shown to be associated with the highest rates of abstinence
- ◯ You recommend starting only nicotine replacement therapy because Varenicline and Bupropion are not safe choices given his history of suicidal ideation
- ◯ You recommend starting only nicotine replacement therapy because starting Varenicline or Bupropion with a history of any psychiatric disorder is associated with an increased risk of serious and overall neuropsychiatric events

---

Esperanza needs a cigarette immediately when she wakes up every morning.

What dose of nicotine gum or lozenge is best for her to use?

- ◯ 2 mg
- ◯ 4 mg
- ◯ 1 mg
- ◯ 7 mg

Kishore smoked 1ppd for 25 years and has had great success after 12 weeks of Varenicline, NRT and support groups. He asks you what his next steps should be.

What do you recommend?

- ◯ You congratulate him and tell him that he can stop the medication right away
- ◯ You congratulate him and advise him to continue taking Varenicline for another 12 weeks
- ◯ You congratulate him and advise him to continue taking Varenicline for another 4 weeks

---

QuestionPro

Obinna is interested in using nicotine replacement therapy in his attempts to quit. He opts for a nicotine patch and gum. After prescribing the treatments, you decide to him the proper method of chewing the gum.

What should you say?

- This gum is no different than chewing a piece of bubble gum. Just make sure to spit it out after 30 minutes. Be sure to not drink coffee or carbonated drinks before or during this process
- Chew the gum until you get a tingling sensation and then place the gum in your cheek until that feeling goes away. Then chew the gum and repeat this process for 30 minutes. Be sure to not drink coffee or carbonated drinks before or during this process
- Chew the gum for 5 minutes then place it in your buccal mucosa for 5 more minutes
- Chew the gum until you get a tingling sensation and then place the gum in your cheek until that feeling goes away. Then chew the gum and repeat this process for 10 minutes

---

Saoirse has a history of coronary artery disease with a CABG in 2012 and is now medically managed. She has been smoking a pack of cigarettes for 30 years and would like to quit.

What do you tell her?

- Varenicline should be avoided because of increased risk for major adverse cardiovascular events and hospitalization for unstable angina
- Bupropion should be avoided because of increased risk for major adverse cardiovascular events and hospitalization for unstable angina
- Nicotine replacement therapy should be avoided because of increased risk for major adverse cardiovascular events and hospitalization for unstable angina
- Varenicline, Bupropion and Nicotine replacement therapy are not associated with increased risk of major adverse cardiovascular events and hospitalization for unstable angina. They are all possible options in patients with stable cardiovascular disease

---

According to the USPSTF, there is insufficient evidence regarding pharmacological interventions in pregnancy. True or False?

- True

○ False

QuestionPro
