# Supplementary material for: Text Message (SMS) Microlearning for Tobacco Use Disorder: Pre-Post Pilot Study of Clinician Confidence
Source: JMIR Med Educ. 2025 Dec 9;11:e73821. doi: 10.2196/73821 (PMC12688373; doi:10.2196/73821)
Supplement: Multimedia Appendix 2 [file mededu-v11-e73821-s002.docx]

Text messages:

Text 1:

Thank you for participating in our smoking cessation text series. On a scale of 1-100, please rate your current level of confidence in treating tobacco use disorder.

Text 2:

Your response has been recorded. Did you know that brief counseling by healthcare providers can increase the chances of smoking cessation? Studies show approximately 1.6 times increased odds of quitting when brief counselling is performed. The Centers for Medicaid and Medicare Services (CMS) recommend a minimum counseling time of 3 minutes, which providers can bill for. (1-2)

Text 3:

In our survey, only 32% of participants correctly identified the recommended first-line pharmacological treatment for tobacco use disorder. When beginning pharmacological treatments for smoking cessation, the following are the recommended options in the order of efficacy:

1. Varenicline in combination with nicotine patch.
2. Combination nicotine replacement therapy (NRT) or Varenicline.
3. Bupropion, which is considered less effective than NRT and Varenicline.

Combining behavioral and pharmacological therapy from the start is recommended to improve success rates in quitting smoking. (3-5)

Text 4:

In our survey, only 58% of participants showed an understanding of Bupropion's contraindications. Bupropion is a viable alternative for patients who cannot take Varenicline or combination nicotine replacement therapy. This atypical antidepressant affects neurotransmitter levels, reducing nicotine cravings and withdrawal symptoms. However, it's not suitable for everyone. Keep in mind that a history of seizures and recent use of MAO inhibitors (within 14 days) are contraindications for starting Bupropion. (6)

Text 5:

In our survey, only 39% of participants correctly selected the nicotine patch dosing based on the number of cigarettes smoked per day. Nicotine patch dosing can be simplified with one question: How many cigarettes does your patient smoke daily?

- If more than 10 cigarettes/day, start with 21mg/day.
- If 10 or fewer cigarettes/day, start with 14mg/day.

Patients can continue the initial dosing for 6 weeks, then reduce by 7mg every 2 weeks. (7)

Text 6:

In our survey, only 63% of providers correctly chose the nicotine gum dosing. Simplifying the dosing for short-term nicotine replacement agents like gum or lozenge can be based on one question: Do you crave a cigarette within 30 minutes of waking up in the morning?

- If yes, use 4mg.
- If no, use 2mg.

Patients may use one piece of gum every 1-2 hours for the first 6 weeks. Frequency can be gradually reduced use over the next six weeks, for a minimum treatment duration of three months. (8)

Text 7:

In our survey, only 66% of providers understood the correct way to use nicotine gum. Patients should be advised to "chew and park" the gum: chew until a tingling sensation occurs, then park it in the cheeks and gums for 30 minutes. Stress the importance of avoiding food and drinks, especially soda and coffee, for 15 minutes before chewing to prevent adverse effects from rapid nicotine absorption. (8)

Text 8:

In our survey, only 14% of providers correctly identified the treatment modalities recommended for pre-contemplative patients. As per the guidelines of the American Thoracic Society, it is advised to offer varenicline to pre-contemplative patients, as it has the potential to enhance cessation rates and reduce cigarette consumption. (9)

Text 9:

In our survey, only 48% of providers correctly prescribed Varenicline for patients with stable psychiatric conditions. Importantly, evidence shows that Varenicline is safe in such patients without active suicidal or homicidal ideation, and the FDA removed the black box warning about potential neuropsychiatric effects in 2016. (10)

Text 10:

In our survey, only 58% of providers correctly identified the recommended duration for Varenicline treatment after a patient quits smoking. Generally, pharmacologic therapy should continue for at least 12 weeks, but it can be extended up to a year based on the patient's risk of relapse. Nicotine Replacement Therapy (NRT) may also be used indefinitely in some cases. (8)

Text 11:

In our survey, 81% of providers knew that pharmacologic therapy doesn't raise the risk of adverse cardiovascular events in stable CAD patients. A large study on stable CVD or CVD risk factors (excluding recent MI/CABG within 2 months) showed no increased risk of major CV events or unstable angina hospitalization after starting varenicline, bupropion, or NRT. (11)

Text 12:

In our survey, only 53% of providers had a clear understanding of the use of pharmacologic interventions for smoking cessation during pregnancy. ACOG and the USPSTF state there's insufficient evidence to evaluate the risks and benefits of pharmacologic therapy in pregnancy. Consequently, behavioral interventions are recommended as the primary approach for smoking cessation during pregnancy. (12-13)

Text 13:

Thank you for joining our smoking cessation text series. On a scale of 1-100, please rate your confidence in treating tobacco use disorder. Did this series increase your comfort level?

Text 14:

Thank you for your response. To access a list of references related to the information provided in this text series, kindly follow the link below.

1. Fiore MC, Bailey WC, Cohen SJ et al. Treating Tobacco Use and Dependence: Clinical Practice Guideline. Rockville, MD: US Department of Health and Human Services, Public Health Service; 2000. http://www.surgeongeneral.gov/tobacco/
2. Silagy C, Stead L. Physician advice for smoking cessation (Cochrane Review) In: The Cochrane Library, Issue 1, 2004. Chichester, UK: John Wiley & Sons, Ltd.
3. [Leone FT, Zhang Y, Evers-Casey S, et al. Initiating Pharmacologic Treatment in Tobacco-Dependent Adults. An Official American Thoracic Society Clinical Practice Guideline. Am J Respir Crit Care Med 2020; 202:e5.](https://www.uptodate.com/contents/pharmacotherapy-for-smoking-cessation-in-adults/abstract/4)
4. [Hajizadeh A, Howes S, Theodoulou A, et al. Antidepressants for smoking cessation. Cochrane Database Syst Rev 2023; 5:CD000031.](https://www.uptodate.com/contents/pharmacotherapy-for-smoking-cessation-in-adults/abstract/16)
5. [Koegelenberg CF, Noor F, Bateman ED, et al. Efficacy of varenicline combined with nicotine replacement therapy vs varenicline alone for smoking cessation: a randomized clinical trial. JAMA 2014; 312:155.](https://www.uptodate.com/contents/pharmacotherapy-for-smoking-cessation-in-adults/abstract/17)
6. Wilkes S. The use of bupropion SR in cigarette smoking cessation. Int J Chron Obstruct Pulmon Dis. 2008;3(1):45-53. doi: 10.2147/copd.s1121. PMID: 18488428; PMCID: PMC2528204.
7. [Hartmann-Boyce J, Aveyard P. Drugs for smoking cessation. BMJ 2016; 352:i571.](https://www.uptodate.com/contents/pharmacotherapy-for-smoking-cessation-in-adults/abstract/71)
8. Centers for Disease Control and Prevention; How to use nicotine gum
9. [Initiating Pharmacologic Treatment in Tobacco-Dependent Adults. An Official American Thoracic Society Clinical Practice Guideline](https://www.atsjournals.org/doi/abs/10.1164/rccm.202005-1982ST)
10. Anthenelli RM, Benowitz NL, West R, St Aubin L, McRae T, Lawrence D, Ascher J, Russ C, Krishen A, Evins AE. Neuropsychiatric safety and efficacy of varenicline, bupropion, and nicotine patch in smokers with and without psychiatric disorders (EAGLES): a double-blind, randomised, placebo-controlled clinical trial. Lancet. 2016 Jun 18;387(10037):2507-20. doi: 10.1016/S0140-6736(16)30272-0. Epub 2016 Apr 22. PMID: 27116918.
11. Rigotti NA, Pipe AL, Benowitz NL, Arteaga C, Garza D, Tonstad S. Efficacy and safety of varenicline for smoking cessation in patients with cardiovascular disease: a randomized trial. Circulation. 2010 Jan 19;121(2):221-9. doi: 10.1161/CIRCULATIONAHA.109.869008. Epub 2010 Jan 4. PMID: 20048210; PMCID: PMC4096941.
12. The American College of Obstetricians and Gynecology; Tobacco and Nicotine Cessation During Pregnancy
13. US Preventive Service Task Force; Tobacco Smoking Cessation in Adults, Including Pregnant Persons: Interventions
